# Chronic obstructive pulmonary disease in rheumatoid arthritis: a systematic review and meta-analysis

**DOI:** 10.1186/s12931-019-1123-x

**Published:** 2019-07-09

**Authors:** Yubo Ma, Hui Tong, Xu Zhang, Mengmeng Wang, Jiajia Yang, Meng Wu, Renfang Han, Mengya Chen, Xingxing Hu, Yaping Yuan, Guixia Pan, Yanfeng Zou, Shengqian Xu, Faming Pan

**Affiliations:** 1 Department of Epidemiology and Biostatistics, School of Public Health, Anhui Medical University, 81 Meishan Road, Hefei, 230032 Anhui China; 2 The Key Laboratory of Major Autoimmune Diseases, Anhui Medical University, 81 Meishan Road, Hefei, 230032 Anhui China; 3 Department of Rheumatism and Immunity, the First Affiliated Hospital of Anhui Medical University, Hefei, 230032 Anhui China

**Keywords:** Rheumatoid arthritis, Pulmonary disease, Chronic obstructive, Prevalence

## Abstract

**Background:**

The risk and prevalence of chronic obstructive pulmonary disease (COPD) in rheumatoid arthritis (RA) is still obscure. The current study was aimed to systematically review and meta-analyse the risk ratio (RR) and prevalence of COPD in RA.

**Methods:**

A comprehensive systematic review was conducted based on PubMed, Web of Science and Cochrane Library from inception to April 30, 2018. The primary outcome of our study was the RR of COPD in RA patients compared with controls, and secondary was the prevalence of COPD in RA patients. Pooled effect sizes were calculated according to fixed effect model or random effects model depending on heterogeneity.

**Results:**

Six and eight studies reported the RR and prevalence of COPD in RA respectively. Compared with controls, RA patients have significant increased risk of incident COPD with pooled RR 1.82 (95% CI = 1.55 to 2.10, *P* <  0.001). The pooled prevalence of COPD in RA patients was 6.2% (95% CI = 4.1 to 8.3%). Meta-regression identified that publication year was an independent covariate negatively associated with the RR of COPD, and smoker proportion of RA population was also positively associated with the prevalence of COPD significantly in RA patients.

**Conclusions:**

The present meta-analysis has demonstrated the significant increased risk and high prevalence of COPD in RA patients. Patients with RA had better cease tobacco use and rheumatologists should pay attention to the monitoring of COPD for the prevention and control of COPD.

**Electronic supplementary material:**

The online version of this article (10.1186/s12931-019-1123-x) contains supplementary material, which is available to authorized users.

## Background

Rheumatoid arthritis (RA) is a common chronic autoimmune disease predominantly affecting synovial joint, and is characterized with joint swell, erosion, pain and mobility limitation [1]. RA usually causes early unemployment, physical disability, reduced life expectancy and mortality, imposing substantial global social burden [2–4]. In addition to joint manifestations, RA patients also frequently accompanied with extra-articular involvements, which significantly increase the morbidity and mortality of RA patients. Studies reported that about half of RA patients will develop respiratory disorders during their life time [5, 6]. Among which, pulmonary diseases were demonstrated as the second leading cause of death of RA patients, account for nearly 20% of the morbidity [7]. Besides the well-known RA-associated interstitial lung disease (ILD) and bronchiectasis, airway diseases were also illustrated to associate with RA recently, such as chronic obstructive pulmonary disease (COPD) [8, 9].

COPD is a chronic progressive inflammatory disease of the distal airways and associated with the chronic inflammation of airways and lung. COPD is characterized with the persistent airflow limitation resulting from inhaling noxious particles and gases [10, 11]. In addition to these risk factors as cigarette smoke, recent studies also reported that the autoimmune response was also contributing to COPD [12–14]. Recently, literatures have reported the pathologic role of autoimmunity in the development of COPD, but the authentic relationship between COPD and autoimmunity diseases, such as RA, was still unclear. Nannini and colleagues first reported that the incidence of COPD in RA patients was significant higher than patients without RA at 2013 [15]. A recent meta-analysis also reminded the tendency of comorbidity of COPD in RA patients, with the limitation of synthesizing the evidence of only four original studies before December 2014 [16]. Since then, new studies have continually reported the relationship between RA and COPD [17, 18]. Besides, meta-analysis focus on the prevalence of COPD in RA patients was still devoid. Therefore, we conducted the present meta-analysis to evaluate the risk and prevalence of COPD in RA patients comprehensively.

## Methods

This meta-analysis was conducted under the guidance of the Preferred Reporting Items for Systematic Reviews and Meta-Analyses (PRISMA) standard [19] (Additional file 1: Table S1) and the Meta-analysis of Observational Studies in Epidemiology (MOOSE) guidelines for systematic reviews of observational studies [20] (Additional file 1: Table S2).

### Review questions

In accordance with the PICOs scheme, the primary review question of present meta-analysis was the RR of COPD (outcome) in RA patients (patient) compared with controls without RA (comparison) of cohort study (study design); the second review question was the prevalence of COPD (outcome) in RA patients (patient) of observational study (study design).

### Search strategies and selection criteria

Two reviewers (Yubo Ma, Hui Tong) independently searched Cochrane Library, PubMed and Web of Science from inception to April 30, 2018 and main search strategy was detailed in the Additional file 1: Figure S1. Besides, pertinent literatures were also manually searched through the references of relevant original studies and reviews. When necessary, the corresponding authors were contacted for full text and detailed data. Eligible studies must respectively fulfill the following included criteria for different outcomes. Primary outcome: a) cohort studies reported the incidence risk of COPD in RA patients compared with controls; b) studies present hazard ratio (HR), risk ratio(RR), standardized incidence ratio (SIR) or original data eligible for the calculate of these indexes. And for the secondary outcome: a) observational studies reported the data of prevalence of COPD in RA; b) selection of RA patients without explicit selection bias. For studies reported duplicate population, the most comprehensive studies contained more participants were included. Additional, all the included studies must written by English.

### Methodological quality assessment and data extraction

Newcastle-Ottawa Scale (NOS) for cohort study and 11-item checklist recommended by Agency for Healthcare Research and Quality (AHRQ) for observational study were applied for the methodological quality assessment of included studies by two investigators (Yubo Ma, Hui Tong) independently. Data about RR and prevalence of COPD were also independently extracted by two reviewers to calculate effect sizes. For the further exploration of the relationship between COPD and RA, the following characteristics were also extracted from included studies: publication year, first author’s name, region of study, study design, the time of study conduct, diagnosis criterion of RA and COPD, disease duration of RA patients and the number, age, gender distribution, follow up duration and smoker proportion of participants. Any discrepancy in processes of literature research, study selection, quality assessment and data extraction was resolved by discussion of the reviewers for consensus.

### Statistical analysis

The pooled RR and prevalence with 95% confidence interval (CI) were calculated to assess the risk and prevalence of COPD in RA patient, through either random effects model or fixed effect model depended on the between studies heterogeneity. Cochran’s Q statistic was used to assess the between studies heterogeneity, and *I*^2^ test was also used complemented to quantify the degree of inconsistency by calculating the percentage of total between studies variation due to heterogeneity rather than chance. Significant between studies heterogeneity was define as the *P* <  0.05 of Q test or *I*^2^ > 50% [21]. The fixed effect model was used where there was no significant heterogeneity, otherwise random effects model was used [22, 23]. Funnel plot, Egger’s linear regression and Begg’s rank correlation test were performed to evaluate publication bias qualitatively and quantitatively [24, 25]. Subgroup analysis for categorical variable and meta-regression for continuous variable were used for exploring potential source of heterogeneity. Sensitivity analysis was also performed to assess the viability of meta-analysis via omitting individual study consecutively. Any *P* <  0.05 was considered statistically significant, and all the statistical analyses were conducted by STATA 11.0 (StataCorp, College Station, TX).

## Results

### Literature research and study characteristics

**Fig. 1:**
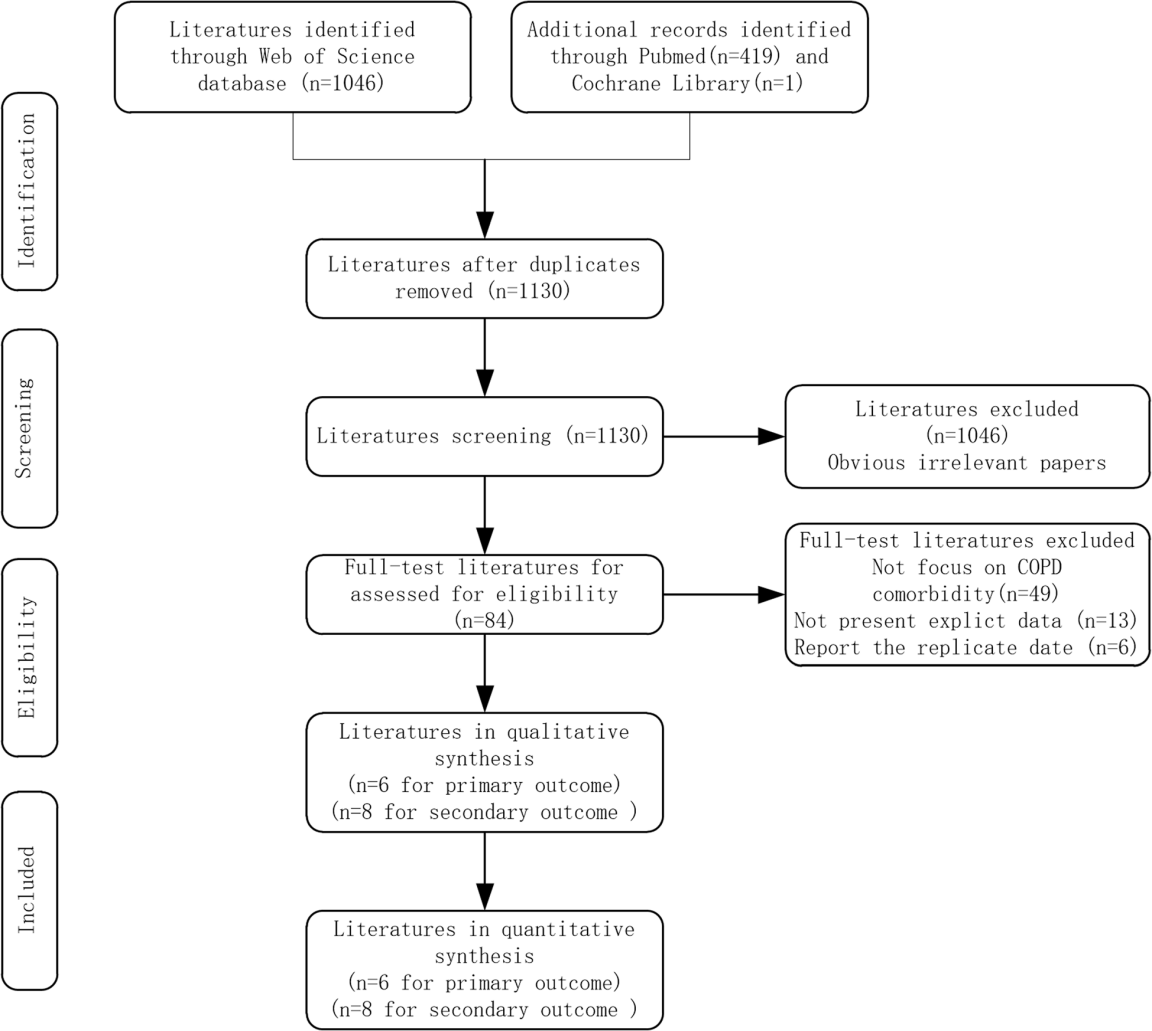
Flow chart of the literature search and study selection

A total of 1466 relevant studies were searched from PubMed (*n* = 419), Web of Science (*n* = 1046) and Cochrane library (n = 1). After the removal of 336 replicate studies and title and abstract screening, 84 studies were retrieved for the full test review. Two studies were removed for reporting data of duplicate population before qualitative synthesis and 14 studies met the inclusion criteria eventually, of which six studies [15, 17, 18, 26–28] reported the risk of COPD and eight studies [2, 29–35] presented the data of the prevalence of COPD in RA (see Fig. 1). All the studies were published between 2010 and 2017, and the methodological quality of included studies were all satisfied, with NOS score among 6 to 9 and AHRQ 11-item checklist scores among 5 to 9. Explicit characteristics of the included studies were represented in Table 1, Table 2 and Table S3 in Additional file 1.

**Table 1:** Characteristics of included studies reported the risk of COPD in RA

Author	Year	Region	Number of cases	Number of controls	Age of cases(Y)	Age of controls(Y)	Female proportion (%)	Follow up duration(Y)	Quality
Sparks JA [17]	2017	America	843	8399	59.8	59.8	100.0	7.0	9
Mcguire K [18]	2017	Canada	24625	25396	57.2	57.3	67.0	18.6	9
Hemminki K [26]	2011	Sweden	NA	NA	NA	NA	NA	NA	6
Nannini C [15]	2013	America	594	596	57.8	58.2	73.4	16.3	9
Ursum J [27]	2013	Netherlands	3356	6708	55.0	55.0	63.7	2.8	8
Shen TC [28]	2014	China	28725	114900	53.8	53.2	78.0	5.1	9

*NA* Not available, *Y* year

**Table 2:** Characteristics of included studies reported the prevalence of COPD in RA

Author	Year	Region	Number of patients	Age (Y)	Female proportion (%)	Smoker proportion (%)	Disease duration (Y)	Quality
Bieber V [29]	2013	Israel	9039	60.1	77.9	28.9	NA	7
Carmona L [30]	2011	Spain	3320	59.0	79.2	14.9	10.3	6
Liao TL [31]	2016	China	42180	53.4	78.6	NA	NA	6
Aurrecoechea E [32]	2016	Spain	140	51.4	50.0	28.6	81.9	5
Lacaille D [33]	2017	Canada	14116	57.5	66.6	NA	0.4	9
Curtis JR [34]	2015	America	13296	52.6	81.9	NA	NA	5
Lunt M [35]	2010	England	16194	57.9	73.4	22.3	9.9	7
Dougados M [2]	2013	Multination	3920	56.0	81.7	13.2	9.6	9

*NA* Not available, *Y* Year

### RR of COPD in RA

**Fig. 2:**
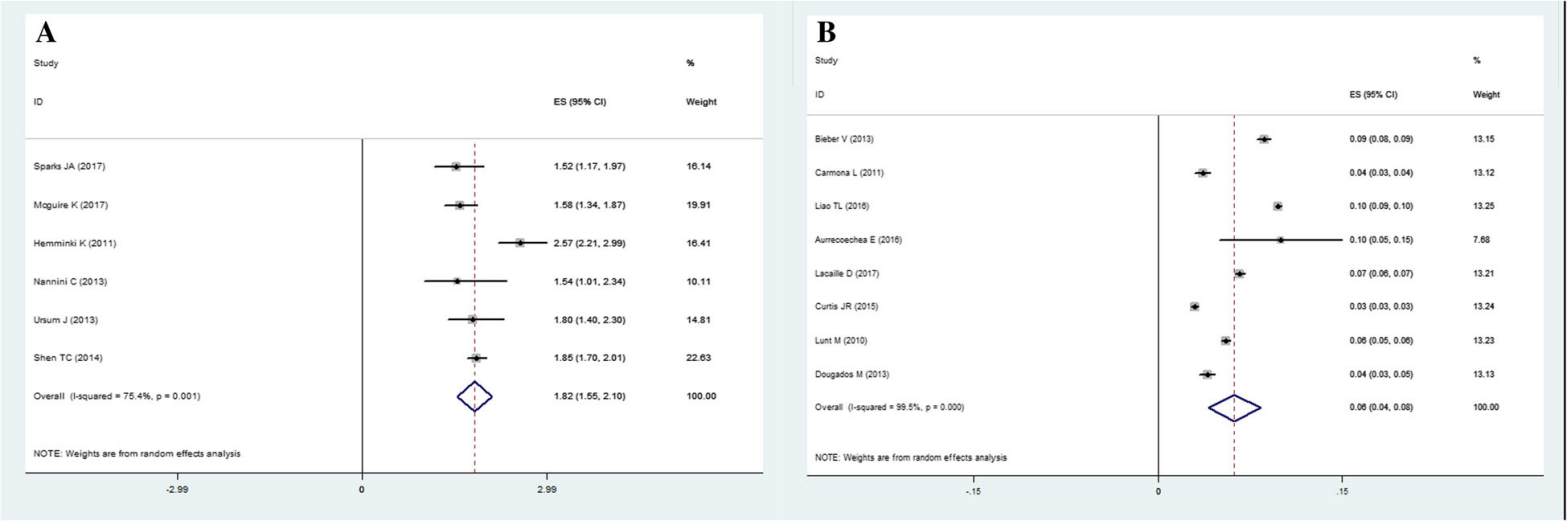
Forest plots of the RR and prevalence of COPD in RA: A) Forest plot of the RR of COPD in RA; B) Forest plot of the prevalence of COPD in RA

Six literatures reported the RR of COPD in RA patients, ranged from 1.52 to 2.57, contained more than 58143 RA patients and 15999 controls. The pooled RR of subsequent incident COPD in RA was 1.82 (95% CI = 1.55 to 2.10, *P* <  0.001) with significant between study heterogeneity (*I*^2^ = 75.4%; Tau^2^ = 0.08, *P* = 0.001) (see Fig. 2). Meta-regression on factors as sample size, age and female proportion of cases, follow up duration and methodological quality indicated that none of them was the source of heterogeneity, but publication year (coefficient = − 0.0772, t = − 4.31, *P* = 0.013, Table 3). Subgroup analysis also reported that RRs of different subgroups were stable according to region of research and diagnosis criteria of RA and COPD detailed in Table 4. By omitting each study consequently, sensitivity analysis found that study by Hemminki and colleagues could explain 66.3% of heterogeneity. After omission, the pooled RR was turned to 1.72 (95% CI = 1.58 to 1.87, *P* <  0.001; *I*^2^ = 18.0%; Tau^2^ = 4.88, *P* = 0.300). Funnel plot of RR was symmetric visually (Additional file 1: Figure S2), with Egger’s linear regression (t = − 0.46, *P* = 0.606) and Begg’s rank correlation (*Z* = 0.38, *P* = 0.707) showing no significant publication bias (Additional file 1: Figure S3).

**Table 3:** Meta-regression analysis coefficients of risk ratio and prevalence of COPD

Variables	Coefficient (SE)	95% CI	t	*P*
Risk ratio				
Publication year	−0.0772 (0.0179)	[− 0.1270,-0.0275]	−4.31	0.013
Age of cases	−0.0387(0.0185)	[− 0.0975, 0.0201]	−2.10	0.127
N of cases	< 0.0001(< 0.0001)	[< −0.0001, 0.0002]	0.87	0.450
N of controls	< 0.0001(< 0.0001)	[< −0.0001, < 0.0001]	1.83	0.164
N of participants	< 0.0001(< 0.0001)	[< −0.0001, < 0.0001]	1.78	0.174
Female proportion	0.0025(0.0019)	[−0.0037, 0.0086]	1.28	0.290
Follow up duration	−0.0112 (0.0066)	[−0.0320, 0.0097]	−1.70	0.187
Quality	−0.2916 (0.1396)	[−0.6793, 0.0962]	−2.09	0.105
Beginning of cohort	−0.0085 (0.0053)	[−0.0232, 0.0062]	−1.61	0.182
Duration of cohort	0.0073 (0.058)	[−.0088, 0.0233]	1.25	0.278
Prevalence				
Publication year	0.0045 (0.0040)	[−0.0052, 0.0143]	1.14	0.296
Number of cases	< 0.0001(< 0.0001)	[< −0.0001, < 0.0001]	1.42	0.206
Age of cases	−0.0011 (0.0036)	[−0.0099, 0.0078]	− 0.30	0.777
Female proportion	−0.0015 (0.0011)	[−0.0042, 0.0012]	−1.36	0.221
Smoker proportion	0.0031 (0.0006)	[0.0012, 0.0049]	5.33	0.013
Disease duration	0.0005 (0.0004)	[−0.0008, 0.0019]	1.32	0.239
Quality	−0.0015 (0.0071)	[−0.0188, 0.0159]	− 0.21	0.844

CI: confidence interval; N: number; SE: standard error

**Table 4:** Subgroup analysis of risk ratio and prevalence of COPD

Subgroups	*N*	Effect size[95% CI]	*Z*	*P*	Test of heterogeneity	
					*I* ^2^	*P*
Risk ratio						
Region						
North America	3	1.560 [1.320, 1.769]	14.58	< 0.001	0.0%	0.968
Europe	2	2.194 [1.439, 2.948]	5.70	< 0.001	84.7%	0.011
Asia	1	1.850 [1.695, 2.005]	23.39	< 0.001	NA	NA
Overall	6	1.822 [1.546, 2.099]	12.92	< 0.001	75.4%	0.001
RA diagnosis						
Database code	3	1.560 [1.320, 1.769]	14.58	< 0.001	0.0%	0.968
Universal criteria	3	2.062 [1.608, 2.517]	8.89	< 0.001	82.9%	0.003
Overall	6	1.822 [1.546, 2.099]	12.92	< 0.001	75.4%	0.001
COPD diagnosis						
Database code	5	1.881 [1.569, 2.192]	11.82	< 0.001	NA	NA
Self-reported	1	1.520 [1.120, 1.920]	7.45	< 0.001	77.7%	0.001
Overall	6	1.822 [1.546, 2.099]	12.92	< 0.001	75.4%	0.001
Prevalence						
Region						
Asia	2	0.092 [0.081, 0.104]	15.78	< 0.001	92.1%	< 0.001
Europe	3	0.052 [0.033, 0.070]	5.58	< 0.001	93.1%	< 0.001
North America	2	0.048 [0.013, 0.084]	2.65	0.008	99.5	< 0.001
Multination	1	0.040 [0.034, 0.046]	12.79	< 0.001	NA	NA
Overall	8	0.062 [0.041, 0.083]	5.74	< 0.001	99.5%	< 0.001
RA diagnosis						
Database code	5	0.067 [0.040, 0.094]	4.86	< 0.001	99.7%	< 0.001
ACR criteria	3	0.041 [0.031, 0.051]	7.90	0.039	69.3%	< 0.001
Overall	8	0.062 [0.041, 0.083]	5.74	< 0.001	99.5%	< 0.001
COPD diagnosis						
Database code	6	0.062 [0.038, 0.086]	4.99	< 0.001	99.6%	< 0.00
NA	2	0.065 [0.007, 0.123]	2.19	0.028	81.8%	0.002
Overall	8	0.062 [0.041, 0.083]	5.74	< 0.001	99.5%	< 0.001

*CI* Confidence interval, *N* Number of study

### Prevalence of COPD in RA

Eight studies containing 102205 RA patients reported the primary prevalence of COPD between 3.0 and 10.0%. Meta-analysis demonstrated that the pooled prevalence of COPD was 6.2% (95% CI = 4.1 to 8.3%) with significant heterogeneity (*I*^2^ = 99.5%; Tau^2^ <  0.01, *P* <  0.001). Likewise, meta-regression illustrated that all the factors of publication year, quality of study and sample size, age, female proportion and disease duration were not the cause of heterogeneity, except smoker proportion (see Table 3). The prevalence of COPD was positively associated with smoker proportion of RA patients (coefficient = 0.0031, t = 5.33, *P* = 0.013). Subgroup analysis demonstrated that the prevalence of different subgroups were consistent. Meanwhile, sensitivity analysis indicated that the pooled effect size was stable whenever any study was omitted. The funnel plot was visually symmetry (Additional file 1: Figure S2) with Begg’s rank correlation (*Z* = 0.37, *P* = 0.711) and Egger’s linear regression (t = − 0.17, *P* = 0.873) (Additional file 1: Figure S4) presenting the absence of publication bias.

## Discussion

Joint destruction and diffused inflammation of RA usually cause reduced quality of life, disability and mortality of patients [2]. Besides, RA related extra-articular manifestations and comorbidities also caused the exacerbated outcome of RA patients. RA-associated ILD was the most well-known pulmonary manifestation with the prevalence around 5–10% [36–38]. Studies reported that ILD and cardiovascular disease are the primary causes of premature death of RA patients, and the 1-year, 5-yaer and 10-year mortality of RA-associated ILD were 13.9, 39.0 and 60.1% [8, 39, 40]. In addition to the pulmonary manifestation, recent studies also reported the increased risk of COPD in RA [17, 18, 41]. Hyldgaard and colleagues also reported the astonished excess burden of COPD in RA patients that the 1-year and 5-year mortalities of RA patients with COPD were 15.0 and 41.9%. Meanwhile, they demonstrated that COPD associated mortality was no less than ILD in RA patients using data derived from identical medical registries and the same methodology [8, 41]. The present analysis reported a statistically significant increased risk of incident COPD with the excess risk of 85%. Potential reasons accounting for the increased risk were present as following.

First, studies proposed that RA and COPD have the similar autoimmune pathogenesis [10, 12, 42, 43]. COPD Patients were also demonstrated to detect autoantibodies to a broad spectrum of self-antigens in serum [43–45]. Patients with genetic and environmental factors susceptible to RA were reported prone to abnormal immune response in pulmonary interstitium and airway [46].

In the other side, studies also reported that smoking may work as a confounding associated with both RA and COPD and result in no-causal association between RA and COPD. It is known that smoking was the main independent risk factor of COPD [47], and tobacco use could increase the predisposition of RA through peptidylarginine deiminases, protein citrullination and the processes of oxidative stress and epigenetic changes. So the more smokers in RA cohort may lead to more COPD in RA patients. Nevertheless, Sparks and colleagues also reported that the RRs of COPD in RA patients were 1.43 adjusted smoking in the entire population and 1.31 in the subset of nonsmokers. Study also reported that smoking cessation of RA patients with COPD would not attenuate the pulmonary inflammation [17, 46].

Third, RA patients usually accompanied with systemic inflammation affecting multiple organs. Long-term chronic inflammation was reported to damage the endothelial cell and cause diverse diseases including atherosclerosis [48]. Likewise, diffuse chronic inflammation in lung of RA may cause lasting destruction of normal construction of pulmonary alveoli and increase the susceptibility to COPD. Therefore, we have reasons to hypothesize that COPD and RA share similar pathogenesis of self reactive immune response, and human comorbidity with RA would increase the risk of concurrent COPD, even smoking may actor as a confounding.

Meanwhile, we have also concluded that the pooled prevalence of COPD among RA patients was 6.2% and positively correlated with the proportion of smokers. In our included studies, three studies reported that the incidence of COPD of RA patients were 2.07, 2.79 and 5.25 per 1000 person-years respectively [18, 26, 28]. Besides, study by Shen also reported that the incidences of subgroups aged 20–34, 35–49, 50–64 and over 65 years were 0.48, 1.09, 4.09 and 17.2 per 1000 person-years [28]. Taken into account of the astonished 1-yaer and 5-year mortality of RA associated COPD, rheumatologist should pay enough attention to the regular monitoring of COPD, especially of aged patients or patients with COPD risk factors like smoking. RA patients should also withdraw tobacco smoking as early as possible not only for the control of disease activity of RA but for the prevention of COPD.

There are still some limitations should be taken into consideration. First, due to the limited information of original studies, only few factors were evaluated to explore the influence on effect size in meta-regression and subgroup analysis, and characteristics, such as smoker proportion of RA cohorts and controls, may work as confounder between RA and COPD and result in inconclusive causal association. Second, still a proportion of the included studies reported the incidence risk and prevalence of COPD based on the data of single medical center but not regional medical data, which would lead to some certain selection bias of patients and inconsistence of results. Third, the present meta-analysis only can provide observational and correlative evidence because of study design and observational nature of original studies. Lastly, significant heterogeneity of the meta-analysis may restrict the generalizability of the results.

## Conclusions

The present meta-analysis has demonstrated the significant increased risk and high prevalence of COPD in RA patients. Rheumatologist should pay attention to the monitoring of COPD in RA patients, and patients had better quit tobacco use as early as possible. Further research is still required for the exploration of cellular and molecular mechanism underlying the association between COPD and RA.

## Additional file

Additional file 1: **Table S1.** PRISMA checklist. **Table S2.** MOOSE checklist. **Table S3.** Detailed characteristics of included studies. **Figure S1.** Search strategy of the electrical databases. **Figure S2.** Funnel plots on the RR and prevalence of COPD in RA: A) Funnel plot on the RR of COPD in RA; B) Funnel plot on the prevalence of COPD in RA. **Figure S3.** Funnel plots of Egger’s linear regression and Begg’s rank correlation test of RR of COPD in RA: A) Begg’s rank correlation; B) Egger’s linear regression. **Figure S4.** Funnel plots of Egger’s linear regression and Begg’s rank correlation test of prevalence of COPD in RA patients: A) Begg’s rank correlation; B) Egger’s linear regression. (DOCX 580 kb)

## Data Availability

Data are available from the authors upon request.

## Abbreviations

AHRQ: Agency for Healthcare Research and Quality
CI: Confidence interval
COPD: Chronic obstructive pulmonary disease
HR: Hazard ratio
ILD: interstitial lung disease
MOOSE: Meta-analysis of Observational Studies in Epidemiology
NOS: Newcastle-Ottawa Scale
PRISMA: Preferred Reporting Items for Systematic Reviews and Meta-Analyses
RA: Rheumatoid arthritis
RR: risk ratio
SIR: Standardized incidence ratio

## References

[1] Scott DL, Wolfe F, Huizinga TW (2010). Rheumatoid arthritis. Lancet..

[2] Dougados M, Soubrier M, Antunez A (2014). Prevalence of comorbidities in rheumatoid arthritis and evaluation of their monitoring: results of an international, cross-sectional study (COMORA). Ann Rheum Dis.

[3] Han GM, Han XF (2016). Comorbid conditions are associated with healthcare utilization, medical charges and mortality of patients with rheumatoid arthritis. Clin Rheumatol.

[4] Ramirez LA, Rodriguez C, Cardiel MH (2015). Burden of illness of rheumatoid arthritis in Latin America: a regional perspective. Clin Rheumatol.

[5] Turesson C, O'Fallon WM, Crowson CS, Gabriel SE, Matteson EL (2003). Extra-articular disease manifestations in rheumatoid arthritis: incidence trends and risk factors over 46 years. Ann Rheum Dis.

[6] Gabbay E, Tarala R, Will R (1997). Interstitial lung disease in recent onset rheumatoid arthritis. Am J Respir Crit Care Med.

[7] Toyoshima H, Kusaba T, Yamaguchi M. [Cause of death in autopsied RA patients]. Ryumachi. 1993;33(3):209–214. 8346462

[8] Hyldgaard C, Hilberg O, Pedersen AB (2017). A population-based cohort study of rheumatoid arthritis-associated interstitial lung disease: comorbidity and mortality. Ann Rheum Dis.

[9] De Soyza A, McDonnell MJ, Goeminne PC (2017). Bronchiectasis rheumatoid overlap syndrome is an independent risk factor for mortality in patients with bronchiectasis: a multicenter cohort study. Chest..

[10] Cosio MG, Saetta M, Agusti A (2009). Immunologic aspects of chronic obstructive pulmonary disease. N Engl J Med.

[11] Miravitlles M, Roche N, Cardoso J (2018). Chronic obstructive pulmonary disease guidelines in Europe: a look into the future. Respir Res.

[12] Wen L, Krauss-Etschmann S, Petersen F, Yu X (2018). Autoantibodies in chronic obstructive pulmonary disease. Front Immunol.

[13] Agusti A, MacNee W, Donaldson K, Cosio M (2003). Hypothesis: does COPD have an autoimmune component?. Thorax..

[14] Sigari N, Moghimi N, Shahraki FS, Mohammadi S, Roshani D (2015). Anti-cyclic citrullinated peptide (CCP) antibody in patients with wood-smoke-induced chronic obstructive pulmonary disease (COPD) without rheumatoid arthritis. Rheumatol Int.

[15] Nannini C, Medina-Velasquez YF, Achenbach SJ (2013). Incidence and mortality of obstructive lung disease in rheumatoid arthritis: a population-based study. Arthritis Care Res (Hoboken)..

[16] Ungprasert P, Srivali N, Cheungpasitporn W, Davis Iii JM (2016). Risk of incident chronic obstructive pulmonary disease in patients with rheumatoid arthritis: a systematic review and meta-analysis. Joint Bone Spine.

[17] Sparks Jeffrey A., Lin Tzu-Chieh, Camargo Carlos A., Barbhaiya Medha, Tedeschi Sara K., Costenbader Karen H., Raby Benjamin A., Choi Hyon K., Karlson Elizabeth W. (2018). Rheumatoid arthritis and risk of chronic obstructive pulmonary disease or asthma among women: A marginal structural model analysis in the Nurses’ Health Study. Seminars in Arthritis and Rheumatism.

[18] McGuire K, Avina-Zubieta JA, Esdaile JM, et al. Risk of incident chronic obstructive pulmonary disease in rheumatoid arthritis: a population-based cohort study. Arthritis Care Res (Hoboken). 2017. 10.1002/acr.23410 29047218

[19] Moher D, Liberati A, Tetzlaff J, Altman DG (2010). Preferred reporting items for systematic reviews and meta-analyses: the PRISMA statement. Int J Surg.

[20] Stroup DF, Berlin JA, Morton SC (2000). Meta-analysis of observational studies in epidemiology: a proposal for reporting. Meta-analysis of observational studies in epidemiology (MOOSE) group. Jama..

[21] Higgins JP, Thompson SG (2002). Quantifying heterogeneity in a meta-analysis. Stat Med.

[22] Higgins JP, Thompson SG, Deeks JJ, Altman DG (2003). Measuring inconsistency in meta-analyses. Bmj..

[23] Borenstein M, Hedges LV, Higgins JP, Rothstein HR (2010). A basic introduction to fixed-effect and random-effects models for meta-analysis. Res Synth Methods.

[24] Egger M, Davey Smith G, Schneider M, Minder C (1997). Bias in meta-analysis detected by a simple, graphical test. Bmj..

[25] Begg CB, Mazumdar M (1994). Operating characteristics of a rank correlation test for publication bias. Biometrics..

[26] Hemminki K, Liu X, Ji J, Sundquist K, Sundquist J (2011). Subsequent COPD and lung cancer in patients with autoimmune disease. Eur Respir J.

[27] Ursum J, Nielen MM, Twisk JW (2013). Increased risk for chronic comorbid disorders in patients with inflammatory arthritis: a population based study. BMC Fam Pract.

[28] Shen TC, Lin CL, Chen CH (2014). Increased risk of chronic obstructive pulmonary disease in patients with rheumatoid arthritis: a population-based cohort study. Qjm..

[29] Bieber V, Cohen AD, Freud T, Agmon-Levin N, Gertel S, Amital HH (2013). Autoimmune smoke and fire-coexisting rheumatoid arthritis and chronic obstructive pulmonary disease: a cross-sectional analysis. Immunol Res.

[30] Carmona L, Abasolo L, Descalzo MA (2011). Cancer in patients with rheumatic diseases exposed to TNF antagonists. Semin Arthritis Rheum.

[31] Liao TL, Lin CH, Chen YM, Chang CL, Chen HH, Chen DY (2016). Different risk of tuberculosis and efficacy of isoniazid prophylaxis in rheumatoid arthritis patients with biologic therapy: a Nationwide retrospective cohort study in Taiwan. PLoS One.

[32] Aurrecoechea E, Llorca Diaz J, Diez Lizuain ML, McGwin G, Calvo-Alen J (2017). Gender-associated comorbidities in rheumatoid arthritis and their impact on outcome: data from GENIRA. Rheumatol Int.

[33] Lacaille D, Avina-Zubieta JA, Sayre EC, Abrahamowicz M (2017). Improvement in 5-year mortality in incident rheumatoid arthritis compared with the general population-closing the mortality gap. Ann Rheum Dis.

[34] Curtis JR, Sarsour K, Napalkov P, Costa LA, Schulman KL. Incidence and complications of interstitial lung disease in users of tocilizumab, rituximab, abatacept and anti-tumor necrosis factor a agents, a retrospective cohort study. Arthritis Res Ther. 2015;17. 10.1186/s13075-015-0835-7 PMC4641344 26555431

[35] Lunt M, Watson KD, Dixon WG (2010). No evidence of association between anti-tumor necrosis factor treatment and mortality in patients with rheumatoid arthritis results from the British Society for Rheumatology biologics register. Arthritis Rheum.

[36] Kelly CA, Saravanan V, Nisar M (2014). Rheumatoid arthritis-related interstitial lung disease: associations, prognostic factors and physiological and radiological characteristics--a large multicentre UK study. Rheumatology (Oxford).

[37] Koduri G, Norton S, Young A (2010). Interstitial lung disease has a poor prognosis in rheumatoid arthritis: results from an inception cohort. Rheumatology (Oxford).

[38] Bongartz T, Nannini C, Medina-Velasquez YF (2010). Incidence and mortality of interstitial lung disease in rheumatoid arthritis: a population-based study. Arthritis Rheum.

[39] Sparks JA, Chang SC, Liao KP (2016). Rheumatoid arthritis and mortality among women during 36 years of prospective follow-up: results from the Nurses' health study. Arthritis Care Res (Hoboken)..

[40] Young A, Koduri G, Batley M (2007). Mortality in rheumatoid arthritis. Increased in the early course of disease, in ischaemic heart disease and in pulmonary fibrosis. Rheumatology (Oxford).

[41] Hyldgaard C, Ellingsen T, Bendstrup E (2018). COPD: an overlooked cause of excess mortality in patients with rheumatoid arthritis. Lancet Respir Med.

[42] Sze MA, Dimitriu PA, Suzuki M (2015). Host response to the lung microbiome in chronic obstructive pulmonary disease. Am J Respir Crit Care Med.

[43] Packard TA, Li QZ, Cosgrove GP, Bowler RP, Cambier JC (2013). COPD is associated with production of autoantibodies to a broad spectrum of self-antigens, correlative with disease phenotype. Immunol Res.

[44] Gamble E, Grootendorst DC, Hattotuwa K (2007). Airway mucosal inflammation in COPD is similar in smokers and ex-smokers: a pooled analysis. Eur Respir J.

[45] Siafakas NM, Tzortzaki EG (2002). Few smokers develop COPD. Why?. Respir Med.

[46] Willis VC, Demoruelle MK, Derber LA (2013). Sputum autoantibodies in patients with established rheumatoid arthritis and subjects at risk of future clinically apparent disease. Arthritis Rheum.

[47] Liu Y, Pleasants RA, Croft JB (2015). Smoking duration, respiratory symptoms, and COPD in adults aged >/=45 years with a smoking history. Int J Chron Obstruct Pulmon Dis.

[48] Manzi S, Wasko MC (2000). Inflammation-mediated rheumatic diseases and atherosclerosis. Ann Rheum Dis.

