# Supplementary material for: Chronic obstructive pulmonary disease in rheumatoid arthritis: a systematic review and meta-analysis
Source: Respir Res. 2019 Jul 9;20:144. doi: 10.1186/s12931-019-1123-x (PMC6617695; doi:10.1186/s12931-019-1123-x)

**Additional file 1:**

**Chronic obstructive pulmonary disease in rheumatoid arthritis: a systematic review and meta-analysis**

**Authors:**

Yubo Ma^a,b,*^, Hui Tong^c,*^, Xu Zhang^a,b,^ , Mengmeng Wang^a,b^, Jiajia Yang^a,b^, Meng Wu^a,b^, Renfang Han^a,b^, Mengya Chen^a,b^, Xingxing Hu^a,b^, Yaping Yuan^a,b^, Guixia Pan^a,b^, Yanfeng Zou^a,b^, Shengqian Xu^c^, Faming Pan^a,b,#^

**Affiliations:**

^a^ Department of Epidemiology and Biostatistics, School of Public Health, Anhui Medical University, 81 Meishan Road, Hefei, Anhui, 230032, China; ^b^ The Key Laboratory of Major Autoimmune Diseases, Anhui Medical University, 81 Meishan Road, Hefei, Anhui, 230032, China; ^c^ Department of Rheumatism and Immunity, the First Affiliated Hospital of Anhui Medical University, Hefei, Anhui, 230032, China.

* Yubo Ma and Hui Tong are contributed equally to this work, and they should be viewed as joint first authors.

**Corresponding Author:**

Faming Pan, Department of Epidemiology and Biostatistics, School of Public Health, Anhui Medical University, 81 Meishan Road, Hefei, Anhui, 230032, China.

E-mail: [famingpan@ahmu.edu.cn](mailto:famingpan@ahmu.edu.cn)

This file presents the supplementary tables and figures, including Systematic Reviews and Meta-Analyses (PRISMA) checklist, Meta-analysis of Observational Studies in Epidemiology (MOOSE) checklist, flow chart of search strategy, funnel plots and funnel plots of Egger’s linear regression and Begg’s rank correlation test.

**Additional Tables**

Additional Table S1. PRISMA checklist

| **Section/topic** | **#** | **Checklist item** | **Reported on page #** |
| --- | --- | --- | --- |
| **TITLE** | | |  |
| Title | 1 | Identify the report as a systematic review, meta-analysis, or both. | 1 |
| **ABSTRACT** | | |  |
| Structured summary | 2 | Provide a structured summary including, as applicable: background; objectives; data sources; study eligibility criteria, participants, and interventions; study appraisal and synthesis methods; results; limitations; conclusions and implications of key findings; systematic review registration number. | 2 |
| **INTRODUCTION** | | |  |
| Rationale | 3 | Describe the rationale for the review in the context of what is already known. | 3 |
| Objectives | 4 | Provide an explicit statement of questions being addressed with reference to participants, interventions, comparisons, outcomes, and study design (PICOS). | 3, 4 |
| **METHODS** | | |  |
| Protocol and registration | 5 | Indicate if a review protocol exists, if and where it can be accessed (e.g., Web address), and, if available, provide registration information including registration number. | - |
| Eligibility criteria | 6 | Specify study characteristics (e.g., PICOS, length of follow-up) and report characteristics (e.g., years considered, language, publication status) used as criteria for eligibility, giving rationale. | 4, 5 |
| Information sources | 7 | Describe all information sources (e.g., databases with dates of coverage, contact with study authors to identify additional studies) in the search and date last searched. | 4 |
| Search | 8 | Present full electronic search strategy for at least one database, including any limits used, such that it could be repeated. | 4, Fig. S1 |
| Study selection | 9 | State the process for selecting studies (i.e., screening, eligibility, included in systematic review, and, if applicable, included in the meta-analysis). | 4, Figure 1 |
| Data collection process | 10 | Describe method of data extraction from reports (e.g., piloted forms, independently, in duplicate) and any processes for obtaining and confirming data from investigators. | 5 |
| Data items | 11 | List and define all variables for which data were sought (e.g., PICOS, funding sources) and any assumptions and simplifications made. | 5 |
| Risk of bias in individual studies | 12 | Describe methods used for assessing risk of bias of individual studies (including specification of whether this was done at the study or outcome level), and how this information is to be used in any data synthesis. | 5, 6 |
| Summary measures | 13 | State the principal summary measures (e.g., risk ratio, difference in means). | 4, 6 |
| Synthesis of results | 14 | Describe the methods of handling data and combining results of studies, if done, including measures of consistency (e.g., I^2^) for each meta-analysis. | 5, 6 |
| Risk of bias across studies | 15 | Specify any assessment of risk of bias that may affect the cumulative evidence (e.g., publication bias, selective reporting within studies). | 5, 6 |
| Additional analyses | 16 | Describe methods of additional analyses (e.g., sensitivity or subgroup analyses, meta-regression), if done, indicating which were pre-specified. | 5, 6 |
| **RESULTS** | | |  |
| Study selection | 17 | Give numbers of studies screened, assessed for eligibility, and included in the review, with reasons for exclusions at each stage, ideally with a flow diagram. | 6, Figure 1 |
| Study characteristics | 18 | For each study, present characteristics for which data were extracted (e.g., study size, PICOS, follow-up period) and provide the citations. | 6, Table 1 and Table 2 |
| Risk of bias within studies | 19 | Present data on risk of bias of each study and, if available, any outcome level assessment (see item 12). | 6, 7 |
| Results of individual studies | 20 | For all outcomes considered (benefits or harms), present, for each study: (a) simple summary data for each intervention group (b) effect estimates and confidence intervals, ideally with a forest plot. | 6, 7, Figure 2, Table 1 and Table 2 |
| Synthesis of results | 21 | Present results of each meta-analysis done, including confidence intervals and measures of consistency. | 6, 7, Figure 2 |
| Risk of bias across studies | 22 | Present results of any assessment of risk of bias across studies (see Item 15). | 6, 7 |
| Additional analysis | 23 | Give results of additional analyses, if done (e.g., sensitivity or subgroup analyses, meta-regression [see Item 16]). | 6, 7, Table 3 and Table 4 |
| **DISCUSSION** | | |  |
| Summary of evidence | 24 | Summarize the main findings including the strength of evidence for each main outcome; consider their relevance to key groups (e.g., healthcare providers, users, and policy makers). | 8-10 |
| Limitations | 25 | Discuss limitations at study and outcome level (e.g., risk of bias), and at review-level (e.g., incomplete retrieval of identified research, reporting bias). | 10 |
| Conclusions | 26 | Provide a general interpretation of the results in the context of other evidence, and implications for future research. | 10 |
| **FUNDING** | | |  |
| Funding | 27 | Describe sources of funding for the systematic review and other support (e.g., supply of data); role of funders for the systematic review. | 11 |

Additional Table S2. MOOSE checklist

| **Item No** | **Recommendation** | **Reported on Page No** |
| --- | --- | --- |
| Reporting of background should include | | |
| 1 | Problem definition | 3, 4 |
| 2 | Hypothesis statement | 3, 4 |
| 3 | Description of study outcome(s) | 3, 4 |
| 4 | Type of exposure or intervention used | 3, 4 |
| 5 | Type of study designs used | 3, 4 |
| 6 | Study population | 3, 4 |
| Reporting of search strategy should include | | |
| 7 | Qualifications of searchers (eg, librarians and investigators) | 4 |
| 8 | Search strategy, including time period included in the synthesis and key words | 4, Figure S1 |
| 9 | Effort to include all available studies, including contact with authors | 4 |
| 10 | Databases and registries searched | 4 |
| 11 | Search software used, name and version, including special features used (eg, explosion) | 4 |
| 12 | Use of hand searching (eg, reference lists of obtained articles) | 4 |
| 13 | List of citations located and those excluded, including justification | 4, Figure 1 |
| 14 | Method of addressing articles published in languages other than English | 5 |
| 15 | Method of handling abstracts and unpublished studies | 5 |
| 16 | Description of any contact with authors | 5 |
| Reporting of methods should include | | |
| 17 | Description of relevance or appropriateness of studies assembled for assessing the hypothesis to be tested | 5 |
| 18 | Rationale for the selection and coding of data (eg, sound clinical principles or convenience) | 5 |
| 19 | Documentation of how data were classified and coded (eg, multiple raters, blinding and interrater reliability) | 5 |
| 20 | Assessment of confounding (eg, comparability of cases and controls in studies where appropriate) | 5 |
| 21 | Assessment of study quality, including blinding of quality assessors, stratification or regression on possible predictors of study results | 5 |
| 22 | Assessment of heterogeneity | 5, 6 |
| 23 | Description of statistical methods (eg, complete description of fixed or random effects models, justification of whether the chosen models account for predictors of study results, dose-response models, or cumulative meta-analysis) in sufficient detail to be replicated | 5, 6 |
| 24 | Provision of appropriate tables and graphics | Figure 1, Figure S1, Figure S3 and Figure S4 |
| Reporting of results should include | | |
| 25 | Graphic summarizing individual study estimates and overall estimate | Figure 2 |
| 26 | Table giving descriptive information for each study included | Table 1 and Table 2 |
| 27 | Results of sensitivity testing (eg, subgroup analysis) | 5-7, Table 3 and Table 4 |
| 28 | Indication of statistical uncertainty of findings | 6, 7 |
| Reporting of discussion should include | | |
| 29 | Quantitative assessment of bias (eg, publication bias) | 6, 7 |
| 30 | Justification for exclusion (eg, exclusion of non-English language citations) | 10 |
| 31 | Assessment of quality of included studies | 7 |
| Reporting of conclusions should include | | |
| 32 | Consideration of alternative explanations for observed results | 10 |
| 33 | Generalization of the conclusions (ie, appropriate for the data presented and within the domain of the literature review) | 10 |
| 34 | Guidelines for future research | 10 |
| 35 | Disclosure of funding source | 10 |

Additional Table S3. Detailed characteristics of included studies

| Author | RA diagnosis criteria | COPD diagnosis criteria | Study characteristics | Study period |
| --- | --- | --- | --- | --- |
| Sparks JA[17] | ACR 1987 or ACR/ELAR 2010 criteria of AS | Self-reported COPD with positive predictive values ranging from 79 -92% | Cohort based on the Nurses’ Health Study, a closed cohort of 121,701 female registered nurses in the United States aged 30-55 years | From June 1, 1976 to 2014 |
| Mcguire K[18] | British Columbia Society of Rheumatology criteria | ICD code | Population based cohort of incident RA cases for the entire province of British Columbia | From January 1996 to March 2010 |
| Hemminki K[26] | ICD code | ICD code | Cohort based on Swedish hospital discharge register | From January 1, 2000 until December 31, 2006 |
| Nannini C[15] | ACR 1987 criteria of RA | Diagnostic code from the database based on medical records | Cohort based on Rochester Epidemiology Project | From January 1, 1955 to January 1, 2006 |
| Ursum J[27] | ICPC code | ICPC code | Cohort based on Dutch Primary Care Database | From 2001 to December 31, 2010 |
| Shen TC[28] | ICD codes | ICD codes | Cohort was based on Taiwan National Health Insurance Research Database | From 1998 to 2010 |
| Bieber V[29] | Diagnostic code from the database based on medical records | Diagnostic code from the database based on medical records | Cross-sectional analysis employed data mining techniques on the CHS health record database | NA |
| Carmona L[30] | ACR 1987 criteria for RA | Diagnostic code from the database based on medical records | Cases from Spanish national drug safety registry and Spanish Society of Rheumatology | NA |
| Liao TL[31] | ICD code | ICD code | Cases from National Health Insurance of Taiwan | NA |
| Aurrecoechea E [32] | ACR 1987 criteria for RA | NA | cross-sectional study performed at Hospital Universitario Sierrallana | NA |
| Lacaille D[33] | ICD code | ICD code | Cases from administrative health data from the entire province of British Columbia | NA |
| Curtis JR[34] | ICD code | ICD code | Cases from MarketScan Commercial Claims and Encounters and the Medicare Supplemental and Coordination of Benefit databases | NA |
| Lunt M[35] | Diagnostic code from the database based on medical records | Diagnostic code from the database based on medical records | Cases from British Society for Rheumatology Biologics Registe | NA |
| Dougados M[2] | ACR 1987 criteria for RA | NA | Nationwide randomized selected cases of France | NA |

ACR: American College of Rheumatology; ELAR: European League Against Rheumatism; ICD: International Classification of Diseases; ICPC: (International Classification of Primary Care)

**Additional Figures**

Additional Fig.S1 Search strategy of the electrical databases


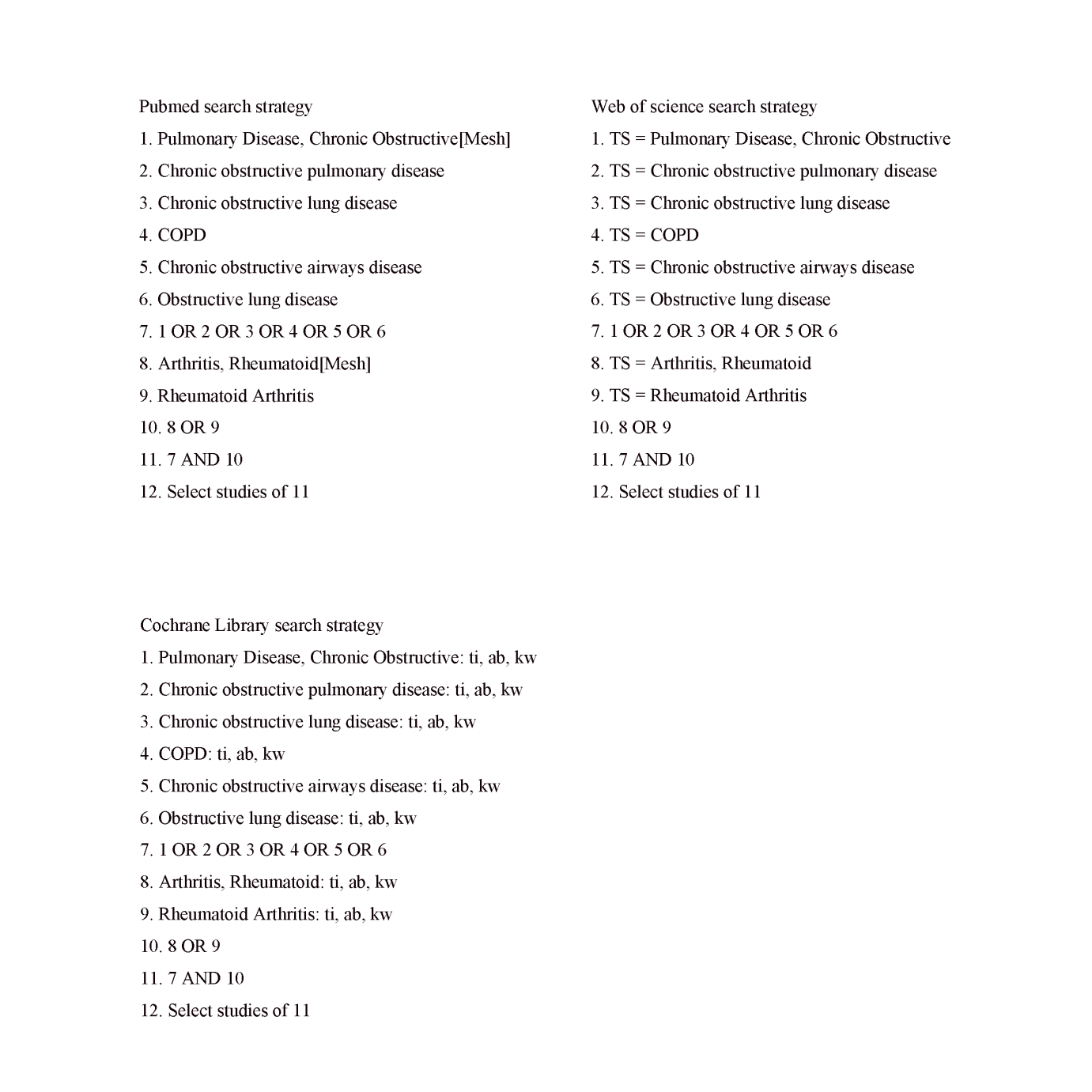


Additional Fig.S2 Funnel plots on the RR and prevalence of COPD in RA: A) Funnel plot on the RR of COPD in RA; B) Funnel plot on the prevalence of COPD in RA


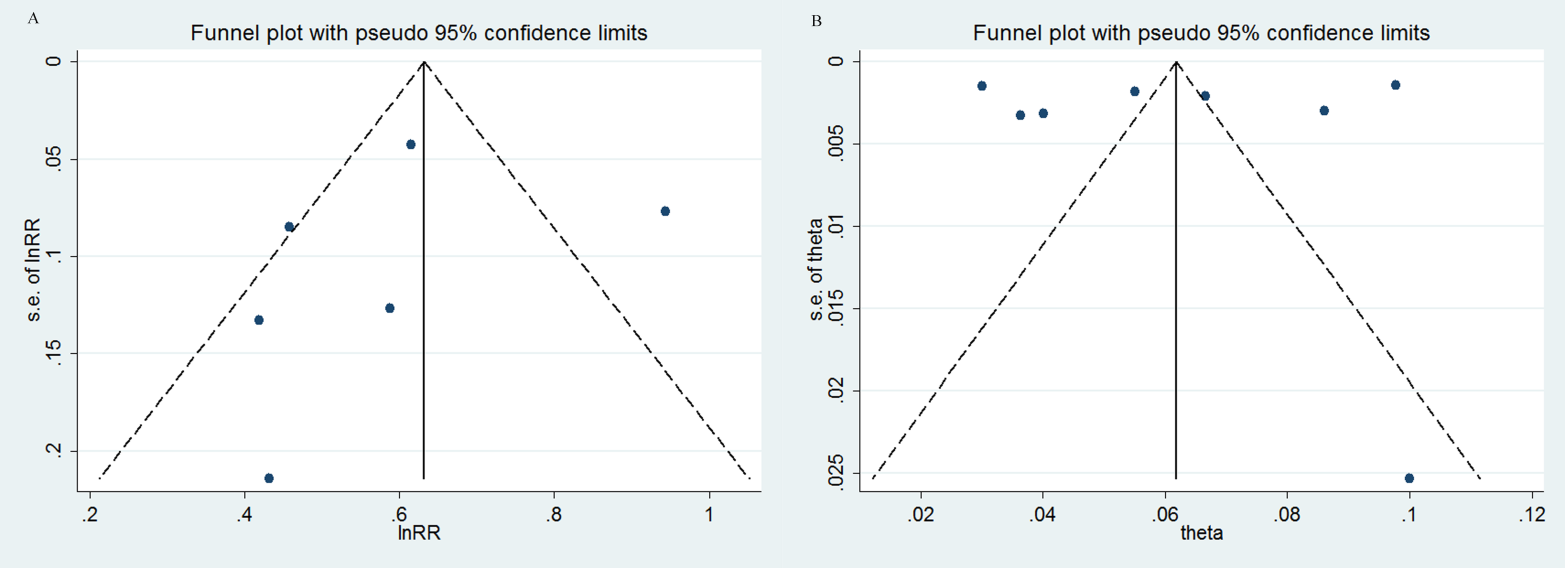


Additional Fig.S3 Funnel plots of Egger’s linear regression and Begg’s rank correlation test of RR of COPD in RA: A) Begg’s rank correlation; B) Egger’s linear regression


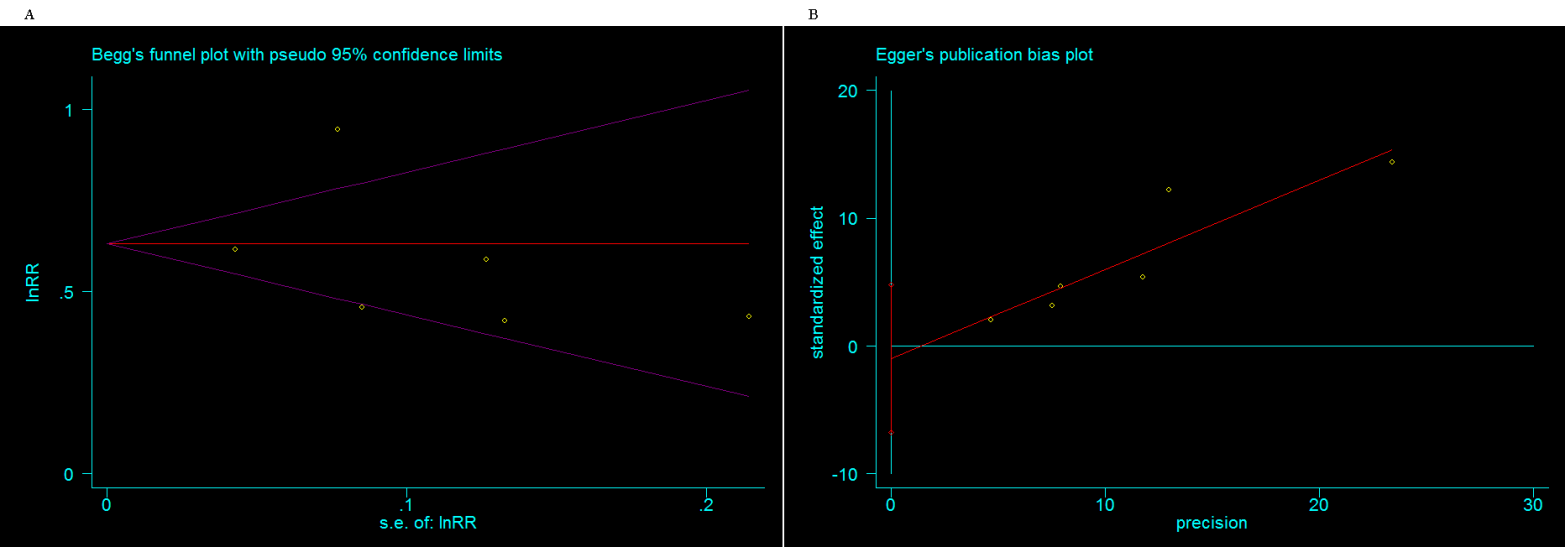


Additional Fig. S4 Funnel plots of Egger’s linear regression and Begg’s rank correlation test of prevalence of COPD in RA patients: A) Begg’s rank correlation; B) Egger’s linear regression


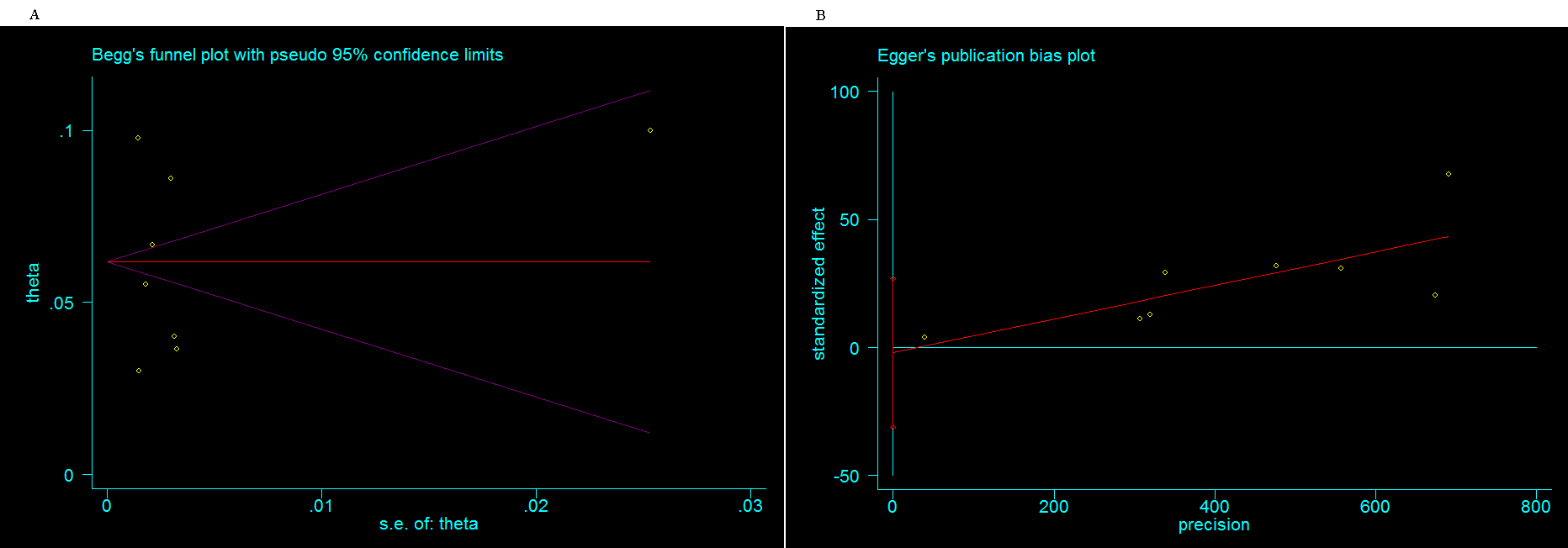

Supplement: Supplementary file 1 — Table S1. PRISMA checklist. Table S2. MOOSE checklist. Table S3. Detailed characteristics of included studies. Figure S1. Search strategy of the electrical databases. Figure S2. Funnel plots on the RR and prevalence of COPD in RA: A) Funnel plot on the RR of COPD in RA; B) Funnel plot on the prevalence of COPD in RA. Figure S3. Funnel plots of Egger’s linear regression and Begg’s rank correlation test of RR of COPD in RA: A) Begg’s rank correlation; B) Egger’s linear regression. Figure S4. Funnel plots of Egger’s linear regression and Begg’s rank correlation test of prevalence of COPD in RA patients: A) Begg’s rank correlation; B) Egger’s linear regression. (DOCX 580 kb) [file 12931_2019_1123_MOESM1_ESM.docx]
